# Prospects of Gels for Food Applications from Marine Sources: Exploring Microalgae

**DOI:** 10.3390/gels11080569

**Published:** 2025-07-23

**Authors:** Antonia Terpou, Divakar Dahiya, Poonam Singh Nigam

**Affiliations:** 1 Department of Agricultural Development, Agri-Food, and Natural Resources Management, School of Agricultural Development, Nutrition & Sustainability, National and Kapodistrian University of Athens, Evripos Campus, 34400 Evia, Greece; 2 Basingstoke and North Hampshire Hospital, Basingstoke RG24 9NA, UK; 3 Faculty of Life & Health Sciences, Ulster University, Coleraine BT52 1SA, UK

**Keywords:** microalgae, gel-forming agents, food hydrocolloids, bioactive compounds, polysaccharides, proteins, natural food stabilizers

## Abstract

The growing demand for sustainable, functional ingredients in the food industry has driven interest in marine-derived biopolymers. Among marine sources, microalgae represent a promising yet underexplored reservoir of bioactive gel-forming compounds, particularly extracellular polysaccharides (EPSs), both sulfated and non-sulfated, as well as proteins that exhibit unique gelling, emulsifying, and stabilizing properties. This study focuses on microalgal species with demonstrated potential to produce viscoelastic, shear-thinning gels, making them suitable for applications in food stabilization, texture modification, and nutraceutical delivery. Recent advances in biotechnology and cultivation methods have improved access to high-value strains, which exhibit promising physicochemical properties for the development of novel food textures, structured formulations, and sustainable food packaging materials. Furthermore, these microalgae-derived gels offer additional health benefits, such as antioxidant and prebiotic activities, aligning with current trends toward functional foods containing prebiotic materials. Key challenges in large-scale production, including low EPS productivity, high processing costs, and lack of regulatory frameworks, are critically discussed. Despite these barriers, advances in cultivation technologies and biorefinery approaches offer new avenues for commercial application. Overall, microalgal gels hold significant promise as sustainable, multifunctional ingredients for clean-label food formulations.

## 1. Introducing Microalgae as a Sustainable Alternative for Food Applications and Gel-Forming Materials

The global population is projected to reach approximately 10 billion by 2050, posing a significant challenge to sustainable food production [1]. In response to this major concern, there is an increasing need for innovative and alternative bioresources. Microalgal biomass is emerging as a valuable resource, rich in both macro- and micro-nutrients. It contains key biopolymers such as polysaccharides, proteins, and lipids, as well as bioactive secondary metabolites like vitamins, carotenoids, phycobiliproteins, polyphenols, and chlorophylls [2]. These components are essential not only for food and nutraceutical applications but also for the development of biopolymers and gel-based materials in various industrial sectors. Likewise, in this new era, the pressing need for scalable, economically viable, and environmentally sustainable gel-forming agents can be met by valorizing algal biomass [3,4,5]. Gels derived from microalgae may offer a renewable and functional alternative to conventional, resource-intensive gelling materials. Moreover, these microalgae-based gels may support the increasing demand for high-quality food products, contributing to a more sustainable and resilient food system.

Today, the microalgae industry is highly dynamic, with numerous new companies entering the market each year. In Europe alone, over 150 companies of various sizes are actively producing microalgae products, predominantly focusing on *Arthrospira platensis* (commonly known as Spirulina) [6,7]. In the United States, the microalgae-based food market is projected to expand significantly, reaching an estimated value of USD 359.87 million by 2032. This growth is driven by major food industry conglomerates investing in research and development to innovate and commercialize microalgae-based food products [8].

Microalgae, including cyanobacteria, have a rich history as a nutritional resource, traditionally consumed for centuries in various regions around the world [9]. Beyond their nutritional value, they are also recognized for their abundant diversity and adaptability, thriving in diverse environments including marine, brackish, and freshwater systems, as well as terrestrial and subaerial habitats [10]. They encompass over 73,000 identified species, though estimates suggest up to 10 million, most of which are autotrophic, while some can grow heterotrophically. Key groups include Cyanobacteria (prokaryotes) and various eukaryotic lineages such as Bacillariophyta, Haptophyta, Ochrophyta, Euglenozoa, Chlorophyta, and Rhodophyta. Despite being distinct, cyanobacteria and microalgae are often grouped due to their shared role as photosynthetic microorganisms [11].

Microalgae like *Arthrospira* (Spirulina), *Chlorella*, and *Aphanizomenon* are widely used for human consumption due to their high protein and nutrient content, while *Dunaliella* and *Haematococcus* are valued for their antioxidant carotenoids. In the food industry, microalgae are incorporated into protein powders, functional drinks, supplements, natural colorants, and antioxidants, offering nutritional and health benefits [9,12]. Beyond human nutrition, microalgae are being increasingly explored and utilized in the circular economy [13,14,15] for their potential in biodiesel production [16], recycling of food industry wastewater through microalgal remediation [17], and as a sustainable source of biomaterials, reflecting their broad utility and commercial value [13,16,17,18].

Today, microalgae are recognized for much more than their traditional use as dietary supplements. Their ease of cultivation and ability to grow under controlled conditions make them an attractive and sustainable source of valuable products [4,19]. Additionally, microalgae and cyanobacteria are increasingly valued for their capacity to generate innovative materials, particularly in the field of hydrocolloids and gel-forming agents, expanding their application potential well beyond traditional uses [4,20,21,22,23].

Recent research demonstrates that polysaccharides and proteins derived from microalgae can be engineered into hydrogels, offering valuable functionalities as texturizers, stabilizers, and controlled-release systems in functional foods [4,5,24,25]. For example, microalgal species like *Porphyridium* and *Nostoc* yield hydrogels suited to biomedical uses [26], while protein-rich algae like Spirulina and Chlorella can be formulated into gels for food technology and therapeutic delivery [3]. These developments also contribute to sustainability goals by reducing reliance on synthetic and animal-derived gelling agents.

To align with the principles of a circular economy, there is a pressing need to adopt novel biorefinery approaches that balance economic viability, social acceptance, and environmental responsibility. This review explores the potential of gels derived from marine microalgae for food applications, focusing on diverse species capable of producing functional gel-forming agents, including polysaccharides and proteins. It examines the mechanisms of gel formation, evaluates the functional and technological properties of these gels, and assesses their current and potential applications in the food industry and beyond. Overall, microalgae-derived gels represent a promising and sustainable alternative to conventional gelling agents, offering significant potential for innovation in future food formulations.

## 2. Microalgae as a Source of Gel-Forming Agents

Several microalgae species show great potential as sources of gel-forming agents, owing to their production of sulfated polysaccharides (SPs), exopolysaccharides (EPSs), and proteins with gel-forming properties (Table 1). *Chlorella* spp., for instance, produce EPSs and proteins that contribute to hydrogel formation, offering texturizing and stabilizing effects in food systems [27]. *Dunaliella* spp. generate polysaccharides that form biofunctional food gels [28], while *A. platensis* yields chitosan-like polysaccharides for active biofilms in food packaging [29]. *A. platensis* protein concentrates, especially in combination with gelatin, are used to produce bioactive films and fast-dissolving nanofibers, supporting aerobic packaging and delivery systems [30,31]. *Nannochloropsis oculata* stands out for its polysaccharide content, enabling hydrogel formation and film development with controlled release capabilities [32]. While *N. oculata* is also a rich source of proteins with nutritional and bioactive potential, these proteins do not currently show gel-forming properties based on scientific evidence [33,34,35]. Its polysaccharide fractions, on the other hand, especially EPSs, are an area of focus for gel-based applications [20]. Nostoc spp., through its high-molecular-weight EPSs, exhibits a robust gel-forming capacity, complemented by *Porphyridium* spp., which contribute EPSs and phycobiliproteins for hydrogels and bioactive films. *Dictyosphaerium chlorelloides* further adds to the portfolio with EPS-driven gel formation [22].

Among the microalgae species, Nostoc commune has been reported to yield approximately 25.7% EPS (dry weight basis) under optimized extraction conditions (100 °C water, four extractions over ~3.5 h) [47]. Its polysaccharide also demonstrates antibacterial activity against *Staphylococcus aureus* and *Escherichia coli* [47]. In comparison, several red microalgal species exhibit exceptionally high polysaccharide yields, making them prime candidates for gel-forming applications. *Porphyridium* sp., the red microalga, produces significant amounts of cell-wall sulfated polysaccharides with strong gel-forming capabilities. Approximately 50–70% of the polysaccharide remains bound to the cell surface, while the rest is secreted into the surrounding medium as a soluble fraction. These polysaccharides exhibit high viscosity and shear-thinning behavior at low concentrations, forming strong, elastic, and reversible gels at around 1% polymer concentration. Due to their exceptional stability under varying pH, salinity, and temperature conditions as well as demonstrated bioactivities such as antioxidant, antiviral, and anti-inflammatory effects, *Porphyridium* polysaccharides have been applied in cosmetics, pharmaceuticals, biolubricants, and as natural food hydrocolloids [52]. For instance, *P. cruentum* and *P. purpureum* both produce polysaccharides exceeding 50% of dry biomass, with moderate protein levels (15–39%), while *Galdieria sulphuraria* offers even higher polysaccharide yields at 63–69% dry weight alongside 26–32% protein [53]. *Dixoniella grisea* contributes 10% cell-bound and 7% released polysaccharides. These sulfated polysaccharides exhibit strong rheological properties, forming viscous, shear-thinning, and reversible gels at low concentrations (~1%), with stability across a wide range of pH and temperature conditions. As a result, these species are highly suitable for use in cosmetics, functional foods, pharmaceuticals, and biolubricant formulations, particularly where bioactivity (e.g., antioxidant, antiviral, or anti-inflammatory effects) is also desired [53].

In contrast, *A. platensis* is best known for its high protein content, reaching up to 50–70% protein of dry biomass under optimized extraction protocols. Its carbohydrate fraction, including exopolysaccharides (EPSs), accounts for 8–14% of dry weight with limited gel-forming capacity. Under optimized cultivation conditions, EPS production can reach ≈97.6 mg/g DW (≈9.8%), and even up to 92.9 mg/g DW (9.3%) when specific nutrient stresses are applied (e.g., high pH or nitrate limitation). These fractions support applications in nutraceutical proteins, bioactive films, and nanofiber-based delivery systems [54].

Similarly, *Chlorella vulgaris* produces moderate amounts of extracellular polysaccharides (EPSs), though less than many red microalgae. For example, Zhang et al. reported EPS concentrations of 0.364 g/L (equivalent to ~5–10% of dry cell weight) when cultivated on BG-11 medium, and 0.208 g/L under standard conditions [55]. Although *C. vulgaris* is easy to cultivate, its relatively low EPS yield results in limited gelling capacity, often requiring blending with other gelling agents to achieve functional hydrogel properties. The EPSs produced by *C. vulgaris* can exhibit shear-thinning behavior, in which viscosity decreases with increasing shear rate, and they can be incorporated into food systems to modify rheological properties and potentially form gels [56]. As such, *C. vulgaris* EPSs are more commonly used in applications such as biosorption and flocculation, rather than as standalone gel-forming agents [57].

**Figure 1:**
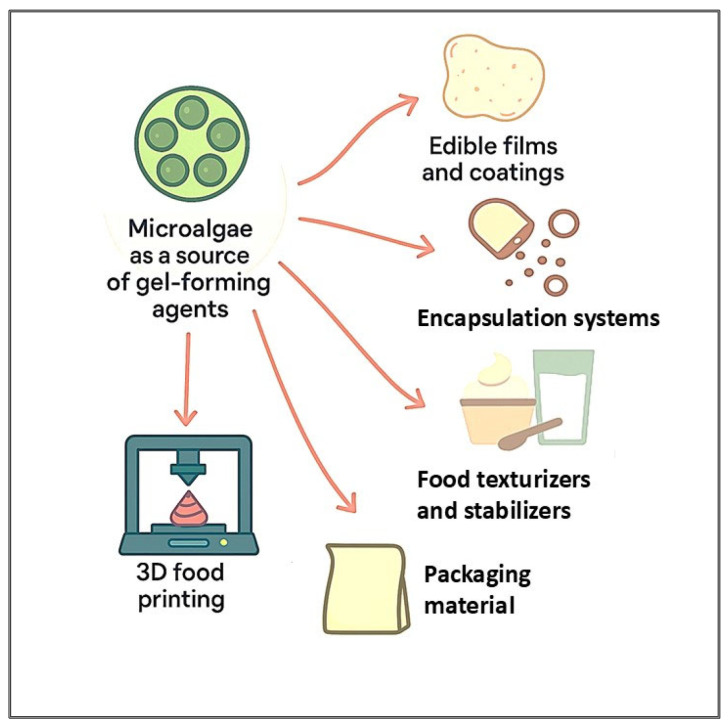
Diverse and promising applications of microalgae-derived gel-forming agents in food, packaging, and biomedical sectors.

Microalgae species collectively demonstrate the versatile and emerging applications of microalgae-derived gel-forming agents in food systems such as edible films and coatings, encapsulation systems, texturizers, and stabilizers as well as in advanced technologies like 3D food printing and biodegradable packaging materials. As illustrated in Figure 1, their functionality supports sustainable innovation across the food [3,57,58,59,60], packaging [8,11,61], and biomedical sectors [8,14,19]. Specifically, in the food industry, microalgal gels can be utilized as natural thickeners, stabilizers, and texturizers in dairy alternatives, desserts, sauces, and dressings, enhancing texture and moisture retention while contributing to clean-label formulations. Their bioactivity and film-forming properties also enable the creation of edible coatings and encapsulation systems for controlled release of nutrients and bioactives, improving product functionality and shelf life. In packaging, microalgae-derived hydrogels and films offer biodegradable, eco-friendly alternatives to petroleum-based materials, aligning with circular economy principles. Additionally, in biomedical applications, these gels exhibit potential for wound healing, tissue engineering, and drug delivery systems due to their biocompatibility, antioxidant, and antimicrobial properties. This multifunctionality underscores the potential of microalgal EPSs and protein-based gels to drive innovation across multiple industries [24,62,63].

### 2.1. Sulfated Polysaccharides (SPs) as Components of Gel Formation

**Figure 2:**
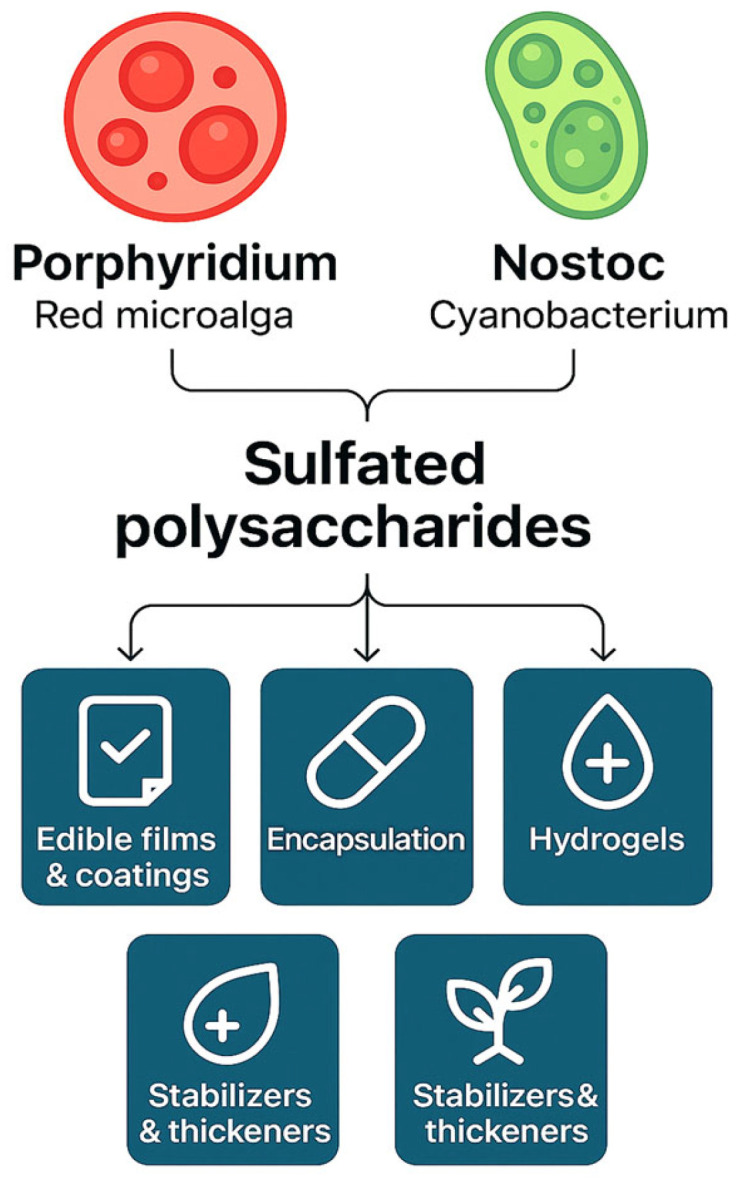
Potential applications of sulfated polysaccharides (SPs) derived from microalgae as bioactive gel-forming agents.

The natural role of polysaccharide biopolymers in microalgal cells is protection against harmful environmental conditions [22,24]. SPs are complex carbohydrates composed of sugar units (monosaccharides) that are modified by the addition of sulfate groups. These sulfate groups give SPs a highly negative charge and unique physicochemical properties. In microalgae, particularly red species, SPs such as carrageenans and agarans are known for their gelling and thickening properties. SPs from red microalgae such as *Porphyridium* spp. and cyanobacteria, including *Nostoc* spp., have attracted significant attention for their unique chemical structures and bio-functional properties (Figure 2). They present a unique and promising source of new hydrogel materials, offering an alternative to widely used polysaccharides such as alginate, carrageenan, and hyaluronic acid [32,49].

Recent studies have revealed that combining sulphated polysaccharides from three red microalgae species with chitosan forms hydrogels with the greatest stiffness and stability observed in the seawater-derived variant, ideal for biomedical use [4]. One of the primary characteristics of these exopolysaccharides is their rheological behavior: they form highly viscous solutions even at relatively low polymer concentrations, and this property is maintained across a broad range of pH values and temperatures, with rheological properties comparable to those of industrial polysaccharides [63]. *Porphyridium* species produce high-molecular-weight SPs, often exceeding 3000 kDa, characterized by a complex backbone of rhamnogalactans, enriched with sulfate groups and uronic acids [49]. These polysaccharides exhibit strong anionic properties, enabling the formation of hydrogels through ionic cross-linking and polyelectrolyte interactions, notably with cationic polymers like chitosan [64].

*Porphyridium* SPs exhibit strong water retention, structural stability under physiological conditions, and diverse bioactivities, including antioxidant, antimicrobial, and anti-inflammatory properties, supporting their use in food hydrocolloids, nutraceuticals, and wound healing applications [65,66,67]. Similarly, *Nostoc* spp., a genus of filamentous cyanobacteria, secrete extracellular SPs with high molecular weights and significant sulfation levels. These SPs are heteropolysaccharides containing uronic acids, glucose, galactose, and rhamnose, contributing to their gel-forming capabilities [64]. *Nostoc* SPs have shown promising bioactivities, such as antioxidant and antimicrobial effects, and their gel networks offer structural integrity and stability, essential for biomedical and food applications [34]. Sulfated polysaccharides from *Porphyridium* and *Nostoc* exemplify the potential of microalgal and cyanobacterial sources as sustainable, functional hydrocolloids [62]. They offer a clean-label alternative to conventional gelling agents and can be applied as encapsulation materials or stabilizers in food products [68,69], as hydrogels for biomedical applications [4,46,63], and as edible films and coatings [11] (Figure 2).

### 2.2. Exopolysaccharides as Components of Gel Formation

EPSs are high-molecular-weight polysaccharides secreted by microorganisms, including bacteria, cyanobacteria, and microalgae, into their surrounding environment. EPSs are a major component of extracellular polymeric substances, contributing to biofilm formation. EPSs from microalgae can also be used as food stabilizers and thickeners, as bioactive compounds with immunomodulatory or antioxidant properties in pharmaceuticals, and in environmental applications such as bioremediation [70,71]. EPSs from *Chlorella* and *Arthrospira platensis* (*Spirulina*) have garnered attention for their gel-forming capabilities, offering promising applications in the food, biomedical, and environmental sectors [25].

Species like *Chlorella vulgaris* and *Chlorella pyrenoidosa* produce EPSs rich in monosaccharides such as glucose, galactose, mannose, xylose, arabinose, and rhamnose. These polysaccharides exhibit notable thermal stability, remaining intact up to 240 °C, and possess antioxidant properties attributed to their sulfate and uronic acid content [72]. The rheological behavior of *Chlorella*-derived EPSs, characterized by high viscosity at low concentrations, makes them suitable as thickeners and stabilizers in food formulations [25]. Additionally, their compatibility with biopolymers like chitosan facilitates the formation of hydrogels and biodegradable films, enhancing their applicability in controlled-release systems and eco-friendly packaging materials [25,71].

*Arthrospira platensis* synthesizes EPSs with a complex composition, including neutral sugars (glucose, rhamnose, galactose, xylose, arabinose, mannose), uronic acids (galacturonic and glucuronic acids), and proteins [5,73,74,75]. These EPSs exhibit non-Newtonian, shear-thinning behavior and maintain gel-like properties across various pH levels and temperatures, indicating their potential as rheology modifiers in food systems [76]. Notably, a specific EPS known as calcium-spirulan (Ca-SP) demonstrates unique structural features, contributing to its bioactivities, including antiviral and anticoagulant effects [77]. The gel-forming properties of Spirulina-derived EPSs, combined with their bioactive functions, make them promising components for functional foods, biomedical hydrogels, and sustainable packaging, which is particularly significant in the post-COVID era, where there is an increasing focus on health and environmental sustainability.

Recent studies also highlight the substantial production of exopolysaccharides (EPSs) by *Dictyosphaerium* species, suggesting that these microalgae generate significant quantities of EPSs renowned for their gelling, thickening, and stabilizing properties in the food industry and beyond [22]. The highly branched and complex structures of rhamnogalactans and arabinogalactans, with diverse linkages and O-methyl substitutions, facilitate strong interactions with water, forming stable, hydrated gel networks. EPSs from *Dictyosphaerium chlorelloides*, with similar structural features, likely exhibit comparable gel-forming, thickening, and water-binding properties [22]. This suggests that *Dictyosphaerium* EPSs could be promising candidates for use as natural gelling, thickening, or stabilizing agents in food, cosmetic, and pharmaceutical applications, offering a sustainable alternative to conventional hydrocolloids.

### 2.3. Protein Isolates as Components of Gel Formation

Protein isolates (PIs) are highly concentrated forms of protein that are separated and purified from other components of a food source. They typically contain 90% or more protein by weight, with minimal amounts of carbohydrates, fats, and other non-protein compounds [78,79]. According to recent studies, *Spirulina* PI shows strong potential for gel formation due to its high protein content and functional characteristics. High-solubility isolates exhibit greater cross-linking and structural folding, whereas lower-solubility isolates demonstrate robust foam capacities and foam stability exceeding 90%, linked to stable interfacial adsorption and good emulsifying properties [75,80].

Enhanced cross-linking at elevated pH further reinforces the internal structure of emulsions, increasing viscosity and network formation [80]. These properties make PIs, especially lower-solubility fractions, valuable as foaming agents, emulsifiers, and gel-forming materials in food systems [60]. For instance, a study on the strain *Spirulina* sp. LEB 18 showed protein concentrates and protein isolate with representative contents of 83.9% and 91.3%, presenting high potential for gel formation in food systems [81]. Additionally, PI extracted from *Spirulina* has been used as a functional coating material to encapsulate commercially valuable pigments, such as phycocyanin [82]. Both *Spirulina* and *Chlorella* have also been incorporated into the production of various food products, including gels [3,58] and fermented milk [83], enhancing their functionality and nutritional value.

A significant recent discovery revealed that *Spirulina platensis* PI can effectively serve as a matrix to create xero-templates for delivering live *Lacticaseibacillus rhamnosus* GG probiotic cells [5]. This finding highlights *Spirulina*’s potential for embedding and protecting probiotic cells, offering an alternative to conventional protein isolates like whey and pea protein. Notably, *Spirulina* PI demonstrated a comparable ability to engraft probiotic cells into the wall material, maintaining cell integrity [5]. This breakthrough paves the way for developing functional foods and nutraceuticals that deliver probiotics in a stable, viable form, expanding the possibilities of PIs from microalgae biomass. As a general outcome on PIs, we can note that the low levels of carbohydrates, ash, and lipids in PI extracts make them ideal for producing stable, protein-based hydrogels suitable for functional foods, nutraceuticals, and clean-label products.

### 2.4. Combination of Gel-Formation Components of Microalgae

**Figure 3:**
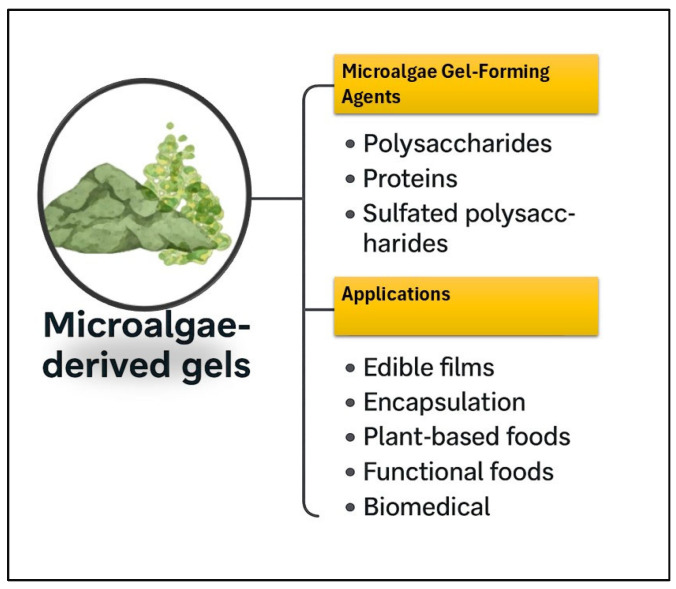
Types of gel-forming agents derived from microalgae and their application potential.

Microalgae provide a sustainable and versatile source of gel-forming agents with significant potential across food, pharmaceutical, and industrial applications [3,25,61,71,80,84,85]. Compounds such as sulfated polysaccharides, exopolysaccharides, and proteins from microalgae demonstrate excellent gel-forming capacity, high water-binding ability, and valuable bioactive properties, including antioxidant and antiviral effects [11,15,26,65,69,71,73,86]. These components contribute to the creation of functional gels, hydrogels, and biofilms, offering solutions for enhanced food texturization, bioactive encapsulation, and the development of sustainable packaging materials (Figure 3). Combining these microalgal compounds with other polymers can yield even more robust and functional gel structures, further broadening their application potential.

A noteworthy emerging concept is the combination of microalgal polysaccharides and proteins with other biopolymers such as chitosan, gelatin, and plant-based gums (e.g., guar gum, xanthan gum, and locust bean gum) [87]. This innovative approach leverages the natural gelling, emulsifying, and stabilizing properties of microalgae-derived compounds while enhancing mechanical strength, elasticity, and barrier properties through synergistic interactions with these complementary biopolymers. For instance, combining sulfated polysaccharides from microalgae with chitosan can create ionic cross-linking networks, resulting in hydrogels with improved gel strength and resilience [4].

Similarly, mixing microalgal proteins with gelatin or plant-based gums enhances the formation of stable, biocompatible, and biodegradable films and gels [31,70]. This method not only expands the functional properties of these biopolymer systems, including texture, water retention, and controlled release capabilities, but also significantly reduces reliance on conventional synthetic polymers (e.g., petroleum-based plastics) and animal-derived ingredients.

Additionally, it aligns with clean-label trends in the food and cosmetic industries, meets circular economy objectives by utilizing renewable biomass, and supports environmental sustainability by lowering carbon footprints and promoting biodegradable material development [10,18]. Such hybrid systems hold potential for diverse applications, including food gels, biomedical hydrogels, edible films, active packaging, and pharmaceutical delivery systems. Ultimately, microalgae-derived gels represent an eco-friendly alternative to conventional gelling materials, supporting progress in global food systems, biomedical technologies, and environmental sustainability.

## 3. Gel-Forming Properties

### 3.1. Molecular Interactions in Gel Formation

Microalgae-derived gel-forming agents rely on several key molecular interactions. Hydrogen bonding is abundant in SPs and EPSs from microalgae, such as *Porphyridium* and *Chlorella*, due to their numerous hydroxyl groups [25]. These hydrogen bonds are crucial for retaining water, maintaining the gel network, and contributing to the gel’s viscoelasticity and stability [88]. For example, red microalgal phycocolloids can form double helices stabilized by hydrogen bonding, which aggregate into gel structures upon cooling [89]. In general, the formation and structure of these networks depend on the polysaccharide’s structural type, the gelling stage, and the concentration of the polysaccharide in solution [89]. Ionic interactions in microalgal SPs arise from their highly negative sulfate groups, enabling cross-linking with divalent cations (e.g., Ca^2+^, Mg^2+^) or positively charged polymers (e.g., chitosan), leading to gels with enhanced strength, thermal stability, and resistance to syneresis, as seen in *Porphyridium* SPs forming ionic bridges with calcium or chitosan [90].

Hydrophobic interactions in microalgal proteins, particularly in *Spirulina* PI, involve the exposure of hydrophobic amino acids during denaturation or pH changes, promoting aggregation and network formation that contribute to gelation and encapsulation of bioactive compounds like phycocyanin [80]. Additionally, combining microalgal SPs or EPSs with proteins or other biopolymers (e.g., chitosan, gelatin) forms hybrid gels or complex coacervates, where hydrogen bonding, ionic interactions, and electrostatic attractions enhance gel integrity, elasticity, and barrier properties, offering advanced functional properties for food, biomedical, and packaging applications [22,24,80].

### 3.2. Role of Environmental Factors in Gel Formation

Environmental factors such as pH, temperature, salinity, and ionic strength play crucial roles in the gel formation and stability of microalgae-derived materials, particularly sulfated polysaccharides (SPs), exopolysaccharides (EPSs), and proteins [78,79,90,91]. The level of pH in solution significantly influences gelation by altering the ionization state of acidic or basic functional groups (e.g., carboxyl, sulfate), affecting electrostatic interactions and hydrogen bonding [92]. For instance, lowering the pH can promote the gelation of SPs by reducing repulsion between negatively charged chains, while proteins like those from *Spirulina* show enhanced aggregation and gelation near their isoelectric point (pH ~ 4.0–5.0), where net charge is minimized [60].

Temperature affects the molecular mobility and hydrogen bond formation, with cooling promoting the gelation of phycocolloids (e.g., agar, carrageenan) from red microalgae by stabilizing double helices [93]. Conversely, heating can denature microalgal proteins, exposing hydrophobic residues and enhancing gel network formation through hydrophobic interactions [79]. Ionic strength and the presence of divalent cations (e.g., Ca^2+^, Mg^2+^) modulate gel formation by neutralizing negative charges on SPs and EPSs, promoting cross-linking and stabilizing gel networks [93,94].

Microalgal gels exhibit optimal gelation in the presence of calcium ions, as these ions enhance cross-linking and network formation [95]. For instance, studies have shown that increasing calcium ion concentrations can initially strengthen gel structures, but beyond certain thresholds, it may cause undesirable aggregation, leading to opacity and decreased gel quality [95]. Similarly, research on alginate gels indicates that while low concentrations of calcium ions increase viscosity and promote gel formation, higher concentrations can lead to the formation of gels with reduced transparency due to aggregation phenomena [96,97]. These findings underscore the importance of optimizing calcium ion concentrations to achieve the desired gel properties in microalgae-derived applications.

Over the past decade, cold plasma technology has emerged as a promising non-thermal approach for enhancing and modifying gel-forming compounds derived from microalgae, particularly extracellular polysaccharides (EPSs) [98]. Cold plasma is widely used for the modification and functionalization of biopolymers due to its ability to alter surface physicochemical properties without the use of solvents or high temperatures [99]. A recent study has demonstrated the potential of cold plasma technology, particularly the plasma discharge system, to enhance the extraction efficiency of phycobiliproteins from *Porphyridium purpureum*, showing superior performance compared to the cold plasma jet approach [100]. In addition, plasma treatment has been shown to structurally modify polysaccharides by reducing their molecular weight, introducing functional groups, and breaking glycosidic bonds. These modifications enhance water solubility, viscosity, and gel-forming behavior. For example, dielectric barrier discharge (DBD) plasma applied to tapioca starch has been reported to lower the molecular weight of amylose and amylopectin, increase sugar content, and reduce viscosity—effects attributed to glycosidic bond cleavage and polymer chain disruption [101]. While most studies focus on plant-derived polysaccharides, similar structural modifications such as bond cleavage, chain scission, and functional group exposure can be expected in microalgal EPSs upon plasma treatment, thereby enhancing their water solubility, shear-thinning behavior, and hydrogel-forming potential.

Overall, precise control of environmental factors such as salinity, light intensity, nutrient availability, temperature, pH, and innovative processing methods like cold plasma is critical for tailoring the physicochemical properties of microalgae-derived gels. These factors influence not only the biosynthesis and secretion of gel-forming compounds such as exopolysaccharides and phycobiliproteins but also their structural attributes, including molecular weight, branching, and functional group composition [32,49,102]. Such variations directly affect the gelation capacity, viscosity, elasticity, and stability of the resulting hydrogels [71,78,90]. In this context, advanced technologies like cold plasma treatment offer a valuable tool for fine-tuning these characteristics post-harvest, expanding the functionality of microalgal gels [98]. Together, environmental modulation during cultivation and targeted post-processing enable the development of application-specific gels, supporting their integration into food, biomedical, and industrial products where clean-label, biodegradable, and bioactive materials are increasingly in demand [98,102]. Continued research into the interplay between cultivation conditions and the structural–functional outcomes of gel-forming agents will be essential to fully unlock the commercial potential of microalgae in gel-based innovations.

## 4. Exploration of Gels from Algal and Non-Algal Sources

Microalgae-derived agents, such as SPs, EPSs, and proteins, demonstrate excellent hydration and water-binding capacity, which is essential for developing hydrogels and biofilms [25,79]. Hydrogen bonding occurs abundantly in SPs and EPSs due to their numerous hydroxyl groups, facilitating hydration, maintaining gel networks, and contributing to gel viscoelasticity and stability; for example, red microalgal phycocolloids form double helices stabilized by hydrogen bonds that aggregate into gel structures upon cooling. Ionic interactions play a crucial role, as the highly negative sulfate groups in SPs enable cross-linking with divalent cations (e.g., Ca^2+^, Mg^2+^) or positively charged polymers like chitosan, resulting in enhanced gel strength, thermal stability, and resistance to syneresis, exemplified by *Porphyridium* SPs forming ionic bridges with calcium or chitosan [62,63,64].

Hydrophobic interactions are prominent in microalgal proteins, such as *Spirulina* PI, where exposure of hydrophobic amino acids during thermal denaturation or pH changes promotes aggregation and network formation, aiding gel structure and the encapsulation of bioactive compounds like phycocyanin. The combination of microalgal SPs or EPSs with proteins or other biopolymers leads to the formation of hybrid gels or complex coacervates, where hydrogen bonding, ionic interactions, and electrostatic attractions enhance gel integrity, elasticity, and barrier properties, providing advanced functionalities [22,24,50]. Table 2 summarizes the use of microalgae for gel-forming agents and end-products.

The overall gel quality and yield from microalgae-derived materials are influenced by factors such as biopolymer composition, extraction methods, polymer concentration, and the use of cross-linkers or co-polymers. Specifically, higher molecular weight, degree of sulfation, and branching in SPs and EPSs improve gel strength and elasticity, while optimized extraction preserves polymer functionality and yield. Increasing polymer concentration enhances gel network density, but excessive levels may induce syneresis. Cross-linkers or co-polymers like chitosan or gelatin introduce additional stabilizing interactions, boosting gel integrity and yield. Post-processing steps like lyophilization and storage conditions further influence gel structure, where improper drying can collapse the network and reduce performance. These insights collectively highlight the scalability and multifunctionality of microalgae-derived gels in diverse applications (Table 2).

Various types of gels can be derived from different biological sources (Table 3). Terrestrial sources such as pectin, gelatin, starch, and plant-based gums are well-established in the food industry, offering predictable, scalable, and functional texturizing and gelling properties [91]. Pectin and gelatin are widely used in jams, jellies, and desserts due to their good gelling capacity at low concentrations and desirable mouthfeel [106]. Plant-derived gums (e.g., gum Arabic, mastic gum) and starch contribute viscosity and stability in sauces, dressings, soft cheese, and bakery and dairy products [106,107]. However, animal-derived agents like gelatin face increasing challenges related to sustainability, ethical sourcing, and consumer acceptance, particularly within vegan, Halal, and Kosher markets. Additionally, these conventional agents generally lack the bioactive functionalities (e.g., antioxidant, antimicrobial, anti-inflammatory) found in microalgae-based gels [7].

Microalgae-derived gels represent an emerging class of biofunctional materials with high market potential, especially in value-added sectors such as pharmaceuticals, nutraceuticals, and active packaging [2,59,61,102]. Compared to conventional gelling agents, microalgal gels offer unique structural diversity, including highly sulfated, branched polysaccharides with tunable viscoelastic properties [3,61]. However, the commercial viability of these gels remains limited by several key production bottlenecks. Large-scale cultivation requires energy-intensive infrastructure (e.g., photobioreactors or raceway ponds), and polysaccharide yields vary significantly depending on the species and culture conditions [17,85]. Furthermore, downstream processing including harvesting, extraction, purification, and drying contributes substantially to operational costs. For instance, medical-grade EPS from *P. purpureum* is estimated to cost around EUR 150/kg at concentrations above 10 g/L [102]. In contrast, the average market price of gelatin ranges from approximately USD 6.7 to 13.3 per kg, depending on the grade and geographic region [108]. Food-grade gelatin typically falls at the lower end of this range, while pharmaceutical and biomedical-grade gelatin commands higher prices due to stricter purity and safety standards [30,70,108]. The global gelatin market has an estimated annual production volume of around 300,000 tonnes and generates between USD 2 and 4 billion in annual revenue, influenced by application sectors and regional demand [108]. Despite its widespread use, gelatin’s animal origin raises sustainability, ethical, and dietary concerns, prompting increased interest in alternative hydrocolloids such as microalgae-derived polysaccharides. While conventional gelling agents are well-established, cost-effective, and scalable, they generally lack the bioactive properties and environmental advantages associated with microalgal gels. The remaining challenge lies in developing sustainable and economically feasible production pathways that can lower the cost of microalgae-derived gel-forming agents and enable broader market adoption.

**Table 3:** Gel-forming agents from various sources and food, biomedical, and packaging applications.

Gel-Forming Agents	Gel-Forming End-Products	References
Pectin (from fruits)	Food gels (e.g., jams, jellies), edible films	[92,109,110]
Gelatin (animal-derived)	Hydrogels, capsules, food gels, foams	[111,112]
Starch (from plants)	Food thickeners, edible films, and biodegradable packaging	[113,114]
Gums (guar, xanthan, mastic, locust)	Stabilizers, thickeners, gels	[115,116,117,118]
Cellulose Derivatives	Hydrogels, thickeners, coatings	[119,120]
Carrageenan (from seaweed)	Food gels, thickening agents, edible films/bioplastics	[121,122,123,124]
Alginate	Encapsulation systems, wound dressings	[94,111,125,126]

From a technological standpoint, microalgae-derived gels offer advantages in clean-label formulations, aligning with current consumer demand for natural, plant-based, and sustainable ingredients. However, scalability and cost remain challenges compared to the well-optimized supply chains for terrestrial agents [59,61,102]. Additionally, the unique ionic cross-linking potential of SPs and the bioactivity of microalgal EPSs make them valuable for designing next-generation functional foods and controlled-release systems. In summary, while terrestrial gel-forming agents provide established functionality and scalability for food texturization, microalgae-derived agents offer innovative biofunctional properties, environmental sustainability, and the potential for enhanced health benefits, positioning them as key players in future functional and bioactive food systems.

## 5. Functional and Technological Properties for Food Applications

**Figure 4:**
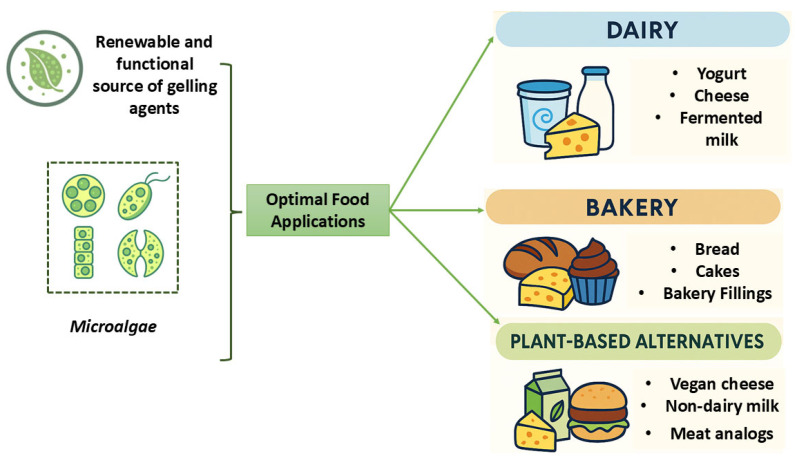
The most prominent food product categories for the potential application of microalgae-derived gel-forming agents.

Microalgae-derived gel-forming agents, particularly extracellular polysaccharides (EPSs) and proteins, are emerging as multifunctional biopolymers with considerable promise in food applications [7,127]. These compounds exhibit a wide array of functional and technological properties, including water-binding capacity, viscosity enhancement, emulsification, and gelation critical for stabilizing emulsions, modifying texture, and structuring diverse food matrices. Among microalgal sources, *Spirulina* and *Chlorella* are especially notable for their ability to form gels, bind water, and alter rheological characteristics. Combined with their bioactive potential, such as antioxidant and immunomodulatory activities, these species are highly attractive for use in functional foods and clean-label formulations [77,83,86,128]. Moreover, dietary fiber derived from microalgae contributes not only to gel formation through hydration and viscoelastic modification but also provides health-promoting benefits, supporting the development of nutritionally enhanced, sustainable food products [30,31,42]. Notably, protein isolates from *Spirulina*, when combined with gelatin, have been successfully processed into functional nanofibers and bioactive films with rapid dissolution and antioxidant and antimicrobial activity, highlighting their relevance for innovative packaging and controlled-delivery systems [31,79]. The primary food sectors where microalgae-derived gel-forming agents demonstrate exceptional promise include dairy, bakery, and plant-based alternatives, where their structural, functional, and nutritional roles align with evolving industry demands (Figure 4). Beyond structural functionality, many microalgal gels contribute additional health-promoting effects, such as antioxidant and prebiotic activity, further enhancing their appeal as clean-label, multifunctional ingredients [79,127]. As interest grows in sustainable and bioactive alternatives to synthetic and animal-derived gelling agents, microalgae offer a unique combination of techno-functional versatility and environmental compatibility [7,18,86,102].

In dairy applications such as yogurt, cheese, and fermented milk, microalgae-derived SPs and EPSs demonstrate excellent compatibility [105,129]. These compounds interact with casein micelles and whey proteins, contributing to enhanced texture, viscosity, and reduction of syneresis (whey separation). Their water-binding and stabilizing properties create a smooth, creamy mouthfeel and improve product shelf life. EPSs, in particular, can increase the viscosity of fermented milk and yogurt systems, making them attractive clean-label alternatives to conventional stabilizers [130]. Optimal functionality is achieved when microalgal compounds are integrated into dairy systems with careful pH and processing condition management to maintain their bioactivity and gel-forming potential [131,132].

In bakery applications, microalgal polysaccharides and proteins function as thickeners and stabilizers in fillings, frostings, and gluten-free doughs [133,134]. These compounds enhance moisture retention and prevent staling, especially in high-protein or gluten-free formulations [134]. Protein isolates from microalgae, such as those derived from Spirulina, can improve the nutritional profile of baked goods and contribute to emulsion stability in batters and doughs. Additionally, microalgal biopolymers support the formation of stable gels in bakery fillings, improving texture and product integrity. Their use aligns with clean-label trends and offers opportunities for formulating healthier, functional bakery products [134].

Although very few studies have directly examined the application of microalgae-derived gel-forming agents in optimizing mouthfeel in steamed foods such as buns or dumplings, evidence from similar moisture-rich systems suggests promising potential for their use in enhancing texture and structural integrity [135]. Exopolysaccharides (EPSs) are recognized for their ability to improve water-holding capacity, viscosity, and gel-forming properties in various food systems—functional attributes that contribute to the structural integrity and softness of moist products such as steamed foods [36,57,136]. Although the direct application of microalgal EPSs in steamed food products is not extensively documented, EPSs extracted from species like Porphyridium and Chlorella have demonstrated hydrocolloid-like behavior in other food matrices, suggesting potential applicability in steamed formulations [26,36,137,138]. Likewise, protein isolates from Spirulina and Chlorella [37,38] have demonstrated emulsifying, foaming, and texturizing properties in foods [5,57,59,75,79,104]. These functional attributes, particularly their capacity to influence structure, elasticity, and water retention, indicate potential applicability in steamed food systems. However, as direct studies on their use in steamed products are currently lacking, further research is warranted to validate their effectiveness and optimize their integration into such moisture-rich food matrices.

In plant-based alternatives such as vegan cheeses, non-dairy milks, meat analogs, and plant-based spreads, microalgae-derived SPs, EPSs, and proteins provide essential gelling, water-binding, and texturizing functions [7,97,134,139]. These agents enhance the structure and mouthfeel of plant-based matrices, mimicking the texture of dairy and meat products. The ionic interactions and gel-forming capacity of SPs and EPSs improve emulsion stability and moisture retention in vegan cheeses and spreads, while microalgal proteins contribute to texture, emulsification, and nutritional enhancement in plant-based beverages and meat analogs. Their compatibility with plant-based ingredients makes them ideal for creating clean-label, sustainable alternatives to conventional animal-derived gelling agents [134].

Despite their valuable bioactive attributes, the broader application of microalgae-derived gel-forming agents remains constrained by several technical and economic challenges. [2,3,61,140]. One significant challenge is the variability in the extraction and purification of sulfated SPs, EPSs, and proteins from microalgal biomass, which can result in inconsistent gel quality and performance [25,79]. The scalability and cost of microalgal production remain limitations compared to conventional terrestrial biopolymers, as large-scale cultivation, harvesting, and processing technologies are still under development and can be cost-prohibitive [102]. To address these issues, the application of innovative technologies such as cold plasma treatment, enzymatic hydrolysis, and cultivation in bioreactors integrated with wastewater bioremediation shows promise in enhancing extraction yields, improving the structural and functional properties of gel-forming compounds, and reducing overall production costs [13,16,23,84,98]. Additionally, optimization of strain selection, process standardization, and formulation strategies such as blending with compatible hydrocolloids could help overcome variability and ensure reproducible gel performance across different food matrices [24,26,59,82,141,142].

Another significant challenge of microalgae-derived gels is the sensory impact, including the potential off-flavors, green color, and odor associated with some microalgal components, which can limit consumer acceptance, especially in delicate food matrices like dairy or bakery products [131]. Microalgae-derived gels often carry distinct sensory traits originating from microalgae, due to their intrinsic biochemical makeup. For instance, *Spirulina platensis* is known to release volatile compounds such as sulfur compounds, aldehydes, ketones, terpenoids, and amines (e.g., trimethylamine and dimethyl sulfide), which impart a strong fishy or earthy odor to extracts and formulations [140,143,144]. Higher processing temperatures such as those used in polymer composite manufacturing can exacerbate odor release, generating ketones and benzene derivatives. Furthermore, the interaction of microalgal volatile compounds with other food ingredients (e.g., proteins, starches, and emulsifiers) may complicate formulation, requiring precise optimization of concentrations and processing conditions [130]. These volatile compounds, identified through gas chromatography–mass spectrometry (GC-MS) analysis, can significantly impact the organoleptic acceptability of final products, particularly when their concentrations exceed the human olfactory threshold, thereby producing perceptible and often undesirable odors that may limit the application of microalgal gels in food and cosmetic formulations [140,144]. Moreover, microalgal pigments such as chlorophyll (green), phycocyanin (blue-green), and carotenoids (red to orange) may remain in partially purified gel extracts, imparting intrinsic coloration that may limit their use in applications requiring transparent or color-neutral formulations [145]. However, this pigmentation is not generally regarded as a safety concern, as these compounds do not adversely interact with the product matrix and may, in fact, provide added functional benefits, including antioxidant and antimicrobial activity, thereby enhancing the bioactivity of the final product without compromising its functional integrity [145,146,147]. Additionally, the presence of natural pigments can yield aesthetically appealing colored gels, which may be favorably perceived by consumers, particularly in food, cosmetic, or nutraceutical applications, where visual appeal is important [11,85,145,148].

In plant-based alternatives, achieving desirable textural and mouthfeel characteristics comparable to animal-derived ingredients is an ongoing challenge, as microalgal gels may not fully replicate the properties of gelatin- or casein-based systems [149]. Regulatory hurdles and limited market familiarity with microalgae-derived ingredients further slow adoption in mainstream food systems. Overcoming these challenges will require advances in microalgal cultivation technology, optimized extraction methods, improved sensory profiling, and clear regulatory pathways to unlock the full potential of microalgae-derived gel-forming agents in diverse food applications.

## 6. Concluding Remarks: Innovations and Emerging Trends for Commercial Potential

Prospects for the usage of microalgae-derived gels in food applications are highly promising, driven by advances in biotechnology, sustainable production methods, and evolving consumer demand for natural and functional ingredients. Innovations such as strain selection, metabolic engineering, and bioreactor optimization are aimed at enhancing the yield, quality, and functional properties of microalgal biopolymers. Additionally, green extraction technologies and hybridization with other biopolymers such as plant-based gums and proteins are expected to generate novel textures and multifunctional food systems.

Microalgae-derived gels, including those from polysaccharides and proteins, offer biodegradable alternatives to synthetic plastics when used as edible films and coatings, while also providing antioxidant and antimicrobial benefits [79,102]. Encapsulation technologies based on microalgal hydrogels enable the delivery of bioactive compounds with enhanced stability and targeted release in functional foods and nutraceuticals. These gels also serve as natural texturizers and stabilizers in plant-based dairy products, desserts, and beverages, contributing to the development of clean-label formulations. Moreover, microalgae-based bioactive hydrogels are showing promise in biomedical fields, particularly for wound healing and tissue engineering applications.

The integration of residual microalgae biomass into zero-waste biorefinery models further enhances the sustainability of gel-forming agent production, particularly for applications in food packaging and related sectors. As the food industry increasingly embraces circular bioeconomy principles, microalgae-derived gels present a promising alternative to synthetic additives, enabling the development of health-promoting, eco-friendly food products [84]. Their structural versatility also aligns well with emerging innovations such as personalized nutrition and 3D food printing, supporting the design of customized textures, nutritional profiles, and targeted health benefits [128,150,151].

Nevertheless, several challenges must be addressed before the full commercialization of microalgae-derived gels can be achieved. A major barrier is production cost, which varies significantly depending on the cultivation method. Open raceway ponds offer lower operational costs, ranging from approximately USD 5.9 to USD 14.2 per kg of dry weight (DW), depending on facility scale, while photobioreactors (PBRs)—despite offering better environmental control—can cost between USD 290 and USD 587 per kg DW due to higher energy and infrastructure demands [152]. Furthermore, downstream processing and extraction must rely on green chemistry approaches to ensure food- and pharmaceutical-grade safety [102].

Variability in microalgae species, cultivation methods, and environmental factors can influence the consistency and functionality of the extracted polysaccharides and proteins, necessitating standardized protocols and optimized bioprocesses. Scaling up production remains a hurdle due to the need for cost-effective cultivation systems, efficient downstream processing, and quality control. Regulatory frameworks governing the use of novel food ingredients, particularly those from marine sources, are still evolving, and clear guidelines are essential to ensure safety and compliance. Consumer acceptance may also be influenced by sensory characteristics, pricing, and perceptions of sustainability. Nonetheless, the growing demand for natural, plant-based, and sustainable food ingredients creates significant opportunities. Partnerships between industry, academia, and regulatory bodies can drive innovation and accelerate product development and approval. Advances in extraction technologies, cross-linking methods, and formulation science will likely unlock the full potential of microalgae-derived gels, positioning them as key components in the next generation of clean-label, functional food products.

## Figures and Tables

**Table 1:** Potential microalgae sources of gel-forming biomolecules.

Microalgae Species	Key Gel-Forming Compound(s)	Major Composition of Gelling Compounds	Gel Applications	References
*Chlorella pyrenoidosa*	Extracellular Polysaccharides (EPSs)	Mannose, Ribose, Rhamnose, Glucuronic acid, Glucose, Galactose, Xylose, Arabinose	Texturizer, stabilizer in food systems	[36]
*Chlorella pyrenoidosa*	Proteins	Edible, structural, and enzymatic proteins	Forming hydrogels	[37,38]
*Dunaliella* spp.	Polysaccharides	Galactose, Glucose, Mannose, Arabinose	Functional food gels with antiviral potential	[28]
*Dunaliella salina*	Extracellular Polysaccharides (EPSs)	Fructose, Galactose, Glucose, Xylose	Food gels	[39]
*Dunaliella tertiolecta*	Extracellular Polysaccharide (EPSs)	α-D-glucan	Limited gelation capabilities compared to complex heteropolysaccharides, unbranched nature	[23]
*Arthrospira platensis* (formerly Spirulina)	Complex heteropolysaccharides (EPSs) known as calcium-spirulan (Ca-SP)	Mannose, Rhamnose, Fructose, Ribose, Galactose, Glucose, Xylose, Galacturonic acid, Glucuronic acid	Viscosity, gelling, and emulsifying properties; supports active biofilms for food packaging and applications in food, cosmetics, and biomedical fields	[29,40,41,42]
*Arthrospira platensis* (formerly Spirulina)	Proteins	Phycobiliproteins: C-phycocyanin (C-PC), Allophycocyanin (APC)	Biofilms, nanofibers, hydrogels, edible films	[30,31,42]
*Nannochloropsis sp.*	Extracellular polysaccharides (EPSs)	Glucose, Mannose, Mannuronic acid, Glucuronic acid, traces of rhamnose and xylose	Biofilm formation	[32,43,44,45]
*Nostoc* spp.	Sulfated polysaccharides/Extracellular Polysaccharides (EPSs)	Glucose, Xylose, Arabinose, Mannose, Galactose, and Uronic acids	Gelling agents with antimicrobial properties	[46,47,48]
*Porphyridium* spp.	Protein	Phycobiliproteins	Reversible gels, biomedical hydrogels	[32,49]
*Porphyridium* spp.	Sulfated polysaccharides/ESPs	Xylose, Galactose, Glucose, Glucuronic acid, and minor amounts of Mannose, Rhamnose, and Arabinose	Viscoelastic gels	[50,51]
*Dictyosphaerium chlorelloides*	Extracellular Polysaccharides (EPSs)	Galactose, Manose, Rhamnose, glucuronic acid	Viscous hydrocolloids	[22]

**Table 2:** Gel-forming agents from microalgae and end-product formation.

Gel-Forming Agents from Microalgae	Gel-Forming End-Products	References
Sulfated Polysaccharides	Hydrogels, Biofilms, Food-gels, Film formation	[4,63,65,69,77,86,103]
Exopolysaccharides	Gel matrices, Encapsulation systems, Stabilizing agents in food	[46,71,73,76]
Protein Isolates	Protein-based gels, Functional coatings, Bioactive films	[11,60,104]
Polysaccharides (non-sulfated)	Food gels with potential bioactivity, Biofilms	[20,21,28,105]
Combined Polymers (e.g., Sulfated Polysaccharides + Chitosan)	Hybrid hydrogels, Enhanced barrier and mechanical properties for packaging	[11,29,30,31,37]

## Data Availability

No new data were created or analyzed in this study.
